# The choroid plexus stroma constitutes a sanctuary for paediatric B‐cell precursor acute lymphoblastic leukaemia in the central nervous system

**DOI:** 10.1002/path.5510

**Published:** 2020-08-28

**Authors:** Lidia M Fernández‐Sevilla, Jaris Valencia, Miguel A Flores‐Villalobos, África Gonzalez‐Murillo, Rosa Sacedón, Eva Jiménez, Manuel Ramírez, Alberto Varas, Ángeles Vicente

**Affiliations:** ^1^ Department of Cell Biology, School of Medicine Complutense University Madrid Spain; ^2^ School of Chemistry Autonomous University of Chihuahua Chihuahua Mexico; ^3^ Department of Paediatric Haematology and Oncology, Advanced Therapies Unit Niño Jesús University Children's Hospital Madrid Spain

**Keywords:** paediatric B‐cell precursor acute lymphoblastic leukaemia, central nervous system, choroid plexus, lymphocytes, haematopoiesis

## Abstract

Despite current central nervous system‐directed therapies for childhood B‐cell precursor acute lymphoblastic leukaemia, relapse at this anatomical site still remains a challenging issue. Few reports have addressed the study of the specific cellular microenvironments which can promote the survival, quiescence, and therefore chemoresistance of B‐cell precursor acute lymphoblastic leukaemia cells in the central nervous system. Herein, we showed by immunofluorescence and electron microscopy that in xenotransplanted mice, leukaemic cells infiltrate the connective tissue stroma of the choroid plexus, the brain structure responsible for the production of cerebrospinal fluid. The ultrastructural study also showed that leukaemia cells are able to migrate through blood vessels located in the choroid plexus stroma. In short‐term co‐cultures, leukaemic cells established strong interactions with human choroid plexus fibroblasts, mediated by an increased expression of *ITGA4* (VLA‐4)*/ITGAL* (LFA‐1) and their ligands *VCAM1/ICAM1*. Upon contact with leukaemia cells, human choroid plexus fibroblasts acquired a cancer‐associated fibroblast phenotype, with an increased expression of α‐SMA and vimentin as well as pro‐inflammatory factors. Human choroid plexus fibroblasts also have the capacity to reduce the proliferative index of leukaemic blasts and promote their survival and chemoresistance to methotrexate and cytarabine. The inhibition of VLA‐4/VCAM‐1 interactions using anti‐VLA‐4 antibodies, and the blockade of Notch signalling pathway by using a γ‐secretase inhibitor partially restored chemotherapy sensitivity of leukaemia cells. We propose that the choroid plexus stroma constitutes a sanctuary for B‐cell precursor acute lymphoblastic leukaemia cells in the central nervous system. © 2020 The Authors. *The Journal of Pathology* published by John Wiley & Sons, Ltd. on behalf of The Pathological Society of Great Britain and Ireland.

## Introduction

B‐cell precursor acute lymphoblastic leukaemia (BCP‐ALL) is the most common form of paediatric cancer and relapsed/refractory disease constitutes the main cause of death by disease in childhood [1, 2, 3, 4]. Leukaemia cells located primarily in the bone marrow egress from there and infiltrate different locations in the body, with a predilection for the central nervous system (CNS). CNS leukaemia is considered to be mainly a leptomeningeal disease, but at late stages disruption of the pial–glial membrane may also provoke ALL invasion of the brain parenchyma, with neural tissue degeneration affecting grey and white matter [5, 6, 7]. Early studies from autopsies of children who died from ALL described CNS involvement in about 60% of cases [6, 8], suggesting that the CNS is already colonized by leukaemic cells at the time of diagnosis. This is also supported by the fact that when the cerebrospinal fluid (CSF) is analysed by flow cytometry and PCR, instead of by microscopic examination, the proportion of CNS‐positive patients rises up to 50% at the time of diagnosis [9, 10, 11]. Moreover, human BCP‐ALL blasts, including those from patients without microscopic evidence of CNS leukaemia, have been reported to mostly be able to infiltrate the CNS in xenograft models [12]. Clonal tracking techniques have shown the polyclonal nature of CNS‐infiltrating leukaemic cells, with multiple clones engrafting in both the CNS and the periphery [12], and CNS leukaemia relapses occur despite the current CNS prophylaxis which includes intrathecal chemotherapy [13]. All of these observations also suggest that some BCP‐ALL cells would be able to survive in particular CNS niches for prolonged periods of time as extramedullary minimal residual disease and could be responsible for CNS relapse.

Information is scant regarding the locations where leukaemic cells could reside in the CNS and for the interactions with cell components of the CNS microenvironment which could induce their quiescence and survival, although increased BCP‐ALL cell survival and chemoresistance to chemotherapeutic agents have been described after co‐culture with cells derived from the meninges, parenchymal astrocytes, and also epithelial cells from the choroid plexus [14, 15, 16]. The choroid plexus (CP) consists of several veil‐like structures projecting into the cerebral ventricles and is constituted by a single layer of epithelial cells, firmly bound by tight junctions, which rests upon a basal lamina and surrounds a central connective tissue stroma where fenestrated capillaries can be found. Functionally, the CP participates in the production and secretion of the CSF; is involved in CNS immune surveillance; and forms one of the brain's barrier interfaces, the blood–CSF barrier (BCSFB) [17, 18]. In the present study, we have shown that BCP‐ALL cells colonize the CP connective tissue stroma and lodge in this location, where the interactions with the microenvironment cell components promote their survival and chemoresistance.

## Materials and methods

### Patient samples

All procedures were performed in accordance with ethical guidelines and regulation, and the study was approved by the Ethics Committee of Clinical Research at Niño Jesús Hospital. Primary samples were obtained from 11 BCP‐ALL patients at diagnosis (supplementary material, Table S1). Routine diagnostic bone marrow aspirates were harvested following standard procedures as required for clinical diagnosis. Leukaemia cells were isolated from marrow aspirates. Samples were provided by the Onco‐Haematology Unit at Niño Jesús University Children's Hospital.

### B‐cell precursor acute lymphoblastic leukaemia xenograft model

NOD.Cg‐Prkdc scid IL2rg tm1Wjl /SzJ (NSG) mice, obtained from Jackson Laboratories (Bar Harbor, ME, USA) and housed under pathogen‐free conditions, were used. All animal experimentation was conducted in accordance with Spanish guidelines for the care and use of laboratory animals, and protocols approved by the Complutense University and Community of Madrid (PROEX 296/15). Three to four 8‐ to 12‐week‐old mice were infused intravenously via the tail vein with 0.5–5 × 10^6^ cells from the different BCP‐ALL samples and engraftment was monitored weekly by peripheral blood cell staining with antibodies against human CD19 (ImmunoStep, Salamanca, Spain) and murine CD45 (BioLegend, San Diego, CA, USA) (supplementary material, Table S3). When disease symptoms including rough hair, lethargy, hunched‐back posture, loss of motor functions, and hind limb paralysis were evident, mice were anaesthetized and transcardially perfused with 0.1 m PBS (pH 7.4) followed by 4% paraformaldehyde. Brains were then removed, fixed, sectioned, and stained.

### Histology

For tissue immunofluorescence analysis, brains were carefully removed, post‐fixed overnight in 4% paraformaldehyde, and placed in 30% sucrose for 48 h. They were then embedded in OCT compound (Thermo Fisher Scientific, Waltham, MA, USA) and frozen at −80 °C before cryostat sectioning (10 μm). Immunofluorescence was performed by blocking sections in 5% normal donkey serum and 0.1% Triton‐X in PBS, and incubating them overnight at 4 °C with primary antibodies against CD19 and pan‐cytokeratin (Sigma, St Louis, MO, USA) (supplementary material, Table S3). After washing with PBS, sections were incubated with the appropriate Alexa Fluor‐conjugated secondary antibodies (Life Technologies, Grand Island, NY, USA). Hoechst 33258 (Molecular Probes, Eugene, OR, USA) was used for nuclear counterstaining. Slides were mounted with Prolong Gold (Life Technologies) and examined using a Nikon Eclipse Ci fluorescence microscope. Image acquisition was performed using Nikon Digital sight DS‐U3 and Nis‐Elements D viewer software. Finally, Fiji software was used for image processing (http://fiji.sc/; last accessed 3 May 2018).

### Electron microscopy

For electron microscopy studies, brains were processed as previously described. In brief, brain samples were fixed by immersion in 4% glutaraldehyde, buffered to pH 7.3 with Millonig's fluid, post‐fixed in 1% osmium tetroxide in the same buffer, and dehydrated in acetone for embedding in Araldite (Sigma). Ultrathin sections were obtained using an OM‐U3 ultramicrotome (Reichert, Vienna, Austria), double‐stained with uranyl acetate and lead citrate, and examined using a JEM 1010 electron microscopy (JEOL, Tokyo, Japan) at the ICTS Spanish National Centre for Electron Microscopy. Images were recorded using a MegaView G2 camera using iTEM Imaging Platform software (Olympus, Münster, Germany).

### Co‐culture assays

The leukaemia BCP‐ALL cell line NALM‐6 (ACC128) was purchased from DSMZ (German Collections of Microorganisms and Cell Cultures, Braunschweig, Germany) and cultured in RPMI 1640 supplemented with 10% FBS, 2 mm l‐glutamine, and penicillin/streptomycin (all from Lonza, Basel, Switzerland). Human choroid plexus fibroblasts were obtained from ScienCell Research Laboratories (Carlsbad, CA, USA) and maintained in the media recommended by the supplier.

Human CP fibroblasts were grown to semi‐confluence on the lower side of 6.5 mm diameter Transwell membranes (0.4 μm pore size; Corning, New York, NY, USA) and NALM‐6 cells were added to the upper compartment of Transwell membranes at a 1:30 ratio and maintained for 12 h in RPMI with 2% FBS. Co‐cultures were also established by directly plating leukaemic cells onto human CP fibroblasts for 24 or 72 h.

The following treatments were used: methotrexate (0.01, 0.1, 1 μm; Pfizer, New York, NY, USA), cytarabine (0.01, 0.1, 1 μm; Pfizer), anti‐VLA‐4 or isotype control antibodies (10 μg/0.5 × 10^6^ cells; BD Biosciences, San José, CA, USA), and γ‐secretase inhibitor DAPT (50 μm; Calbiochem, Nottingham, UK).

### Flow cytometry

Anti‐human CD19, VCAM1, and ICAM1 mAbs conjugated to different fluorochromes (from BioLegend and BD Biosciences) (supplementary material, Table S3) were used for flow cytometry analysis. Immunofluorescence staining was carried out by incubating cells in PBS containing 1% FBS and 0.1% NaN_3_ in the presence of saturating amounts of fluorochrome‐conjugated mAbs for 30 min at 4 °C. To avoid binding of antibodies to Fc receptors, cells were pre‐incubated for 5 min at 4 °C with FcR Blocking Reagent, following the supplier's instructions (Miltenyi Biotec, Bergisch Gladbach, Germany). All analyses were conducted using a FACSCalibur flow cytometer (BD Biosciences) at the Centro de Citometría y Microscopía de Fluorescencia (Complutense University, Madrid, Spain).

For cell cycle analysis, cells harvested from culture plates were fixed and permeabilized with 70% ethanol. After several washes in PBS, 7‐amino‐actinomycin D (7‐AAD; Sigma) was added and the cell cycle analysed using flow cytometry.

### Viability assays

In cell co‐cultures treated with methotrexate or cytarabine, identification and quantification of viable and apoptotic cells were carried out by flow cytometry using annexin V (ImmunoStep, Salamanca, Spain) and propidium iodide, in combination with specific primary antibodies when necessary. Cells were analysed using a FACSCalibur flow cytometer and gated according to forward scatter, side scatter, and their ability to exclude propidium iodide. Viable cells were defined as propidium iodide‐ and annexin V‐negative cells.

### Cytokine measurements

IL‐6, IL‐8, and CCL2 were assayed in supernatants collected from choroid plexus fibroblast/leukaemia cell co‐cultures using commercially available ELISA kits (BioLegend) or the Cytometric Bead Array (CBA) Flex Set system (BD Biosciences), following the manufacturer's recommendations.

### Immunocytofluorescence

Human choroid plexus fibroblasts cultured in chamber slides with or without leukaemia cells were fixed with 4% PFA for 15 min, treated with 0.05% saponin in PBS for 1 h, and then incubated with primary antibodies against the following molecules: CD19, vimentin (Cell Signaling Technology, Danvers, MA, USA), and α‐SMA (Merck Millipore, Burlington, MA, USA) (supplementary material, Table S3) for 1 h at room temperature. After washing, cells were incubated with the appropriate Alexa Fluor‐conjugated secondary antibodies. Hoechst 33258 was used for nuclear counterstaining. Slides were mounted with Prolong Gold and examined using fluorescence microscopy.

### RT‐qPCR

RNA was isolated by using an Absolutely RNA Microprep Kit (Agilent Technologies, Santa Clara, CA, USA) including a DNAse digestion step, as recommended by the supplier, to avoid genomic DNA contamination. Total cDNA was synthesized with the High Capacity cDNA Reverse Transcription Kit (Applied Biosystems, Foster City, CA, USA) according to the manufacturer's instructions and real‐time quantitative PCR was performed by using Taqman assays obtained from Applied Biosystems (supplementary material, Table S2). *GNB2L1* was used as an endogenous control. Amplifications, detections, and analyses were performed using a 7900HT Fast Real‐Time PCR System (Applied Biosystems) at the Centro de Genómica, UCM.

### Statistical analysis

Statistical comparisons were performed with the Mann–Whitney test using GraphPad Prism 8.0 software (GraphPad Inc, San Diego, CA, USA). Values of *p* ≤ 0.05 (*), *p* ≤ 0.01 (**), and *p* ≤ 0.001 (***) were considered to be statistically significant.

## Results

### Acute lymphoblastic leukaemia cells infiltrate the choroid plexus stroma

**Figure 1:**
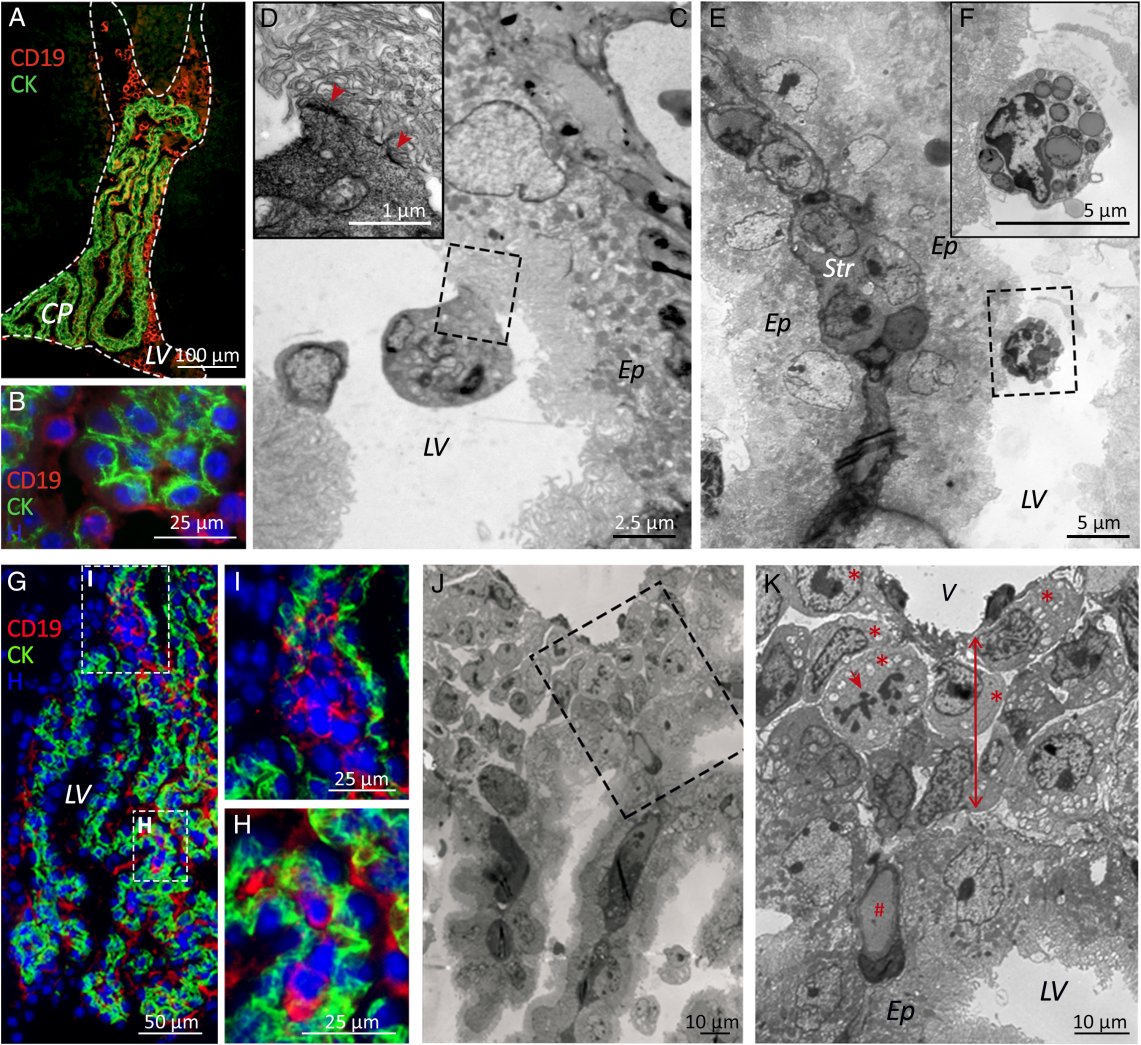
B‐cell precursor acute lymphoblastic leukaemia cells infiltrate the connective stroma of the choroid plexus in a xenograft model. (A) Immunofluorescence microscopy images show BCP‐ALL cells (hCD19 staining, red) floating in the CSF‐filled lateral brain ventricles (LV, dashed lines) and (B) in contact with CP epithelial cells (cytokeratin staining, green). (C, D) Electron microscopy images of leukaemic cells tightly attached (red arrowheads) to the apical surface of the CP epithelium (Ep) and (E, F) Kolmer cells containing CSF‐filled vesicles also in contact with CP epithelial cells. A stromal core of connective tissue (Str) surrounded by two epithelial linings can be seen in E. (G–I) Small groups of BCP‐ALL cells (hCD19 staining, red) located within the CP stroma, (I) mainly in the attachments of the CP. (J, K) Electron micrographs showing leukaemic blasts (red asterisks), some of them in mitosis (red arrow), housed in the space (red two‐sided arrow) between basal laminae of the CP epithelium and CP blood vessels (V). # denotes a CP capillary. Samples from patients 4 and 5 were used for electron microscopy studies, and samples from patients 1–3 and 6–11 for immunofluorescence studies.

Primary BCP‐ALL cells injected into NSG mice were able to invade the CNS and largely accumulated in the subarachnoid space, but could also be detected in the encephalic ventricles usually in contact with the CP (Figure 1A). Immunofluorescence and electron microscopy studies showed that in this location leukaemic cells established tight interactions with the apical surface of the CP epithelium (Figure 1B–D). Sporadically, and attached to the CP epithelial cells, Kolmer cells were also identified, appearing as cells filled of vesicles containing CSF and with no sign of leukaemic cell phagocytosis (Figure 1E,F).

Interestingly, small foci of CD19^+^ leukaemic cells could also be observed in the stroma of the CP, mainly in the CP attachments (Figure 1G–I). Leukaemic blasts, some in mitosis, were lodged in the loose connective tissue appearing between basal laminae of the CP epithelium and the adjacent capillary and venule endothelium (Figure 1J,K). Of note, although BCP‐ALL cells from all samples could be seen in this location, not all mice injected with the same primary cell sample showed evident CP infiltration (range 20–50%). No differences were observed among different primary samples according to risk or site of relapse.

**Figure 2:**
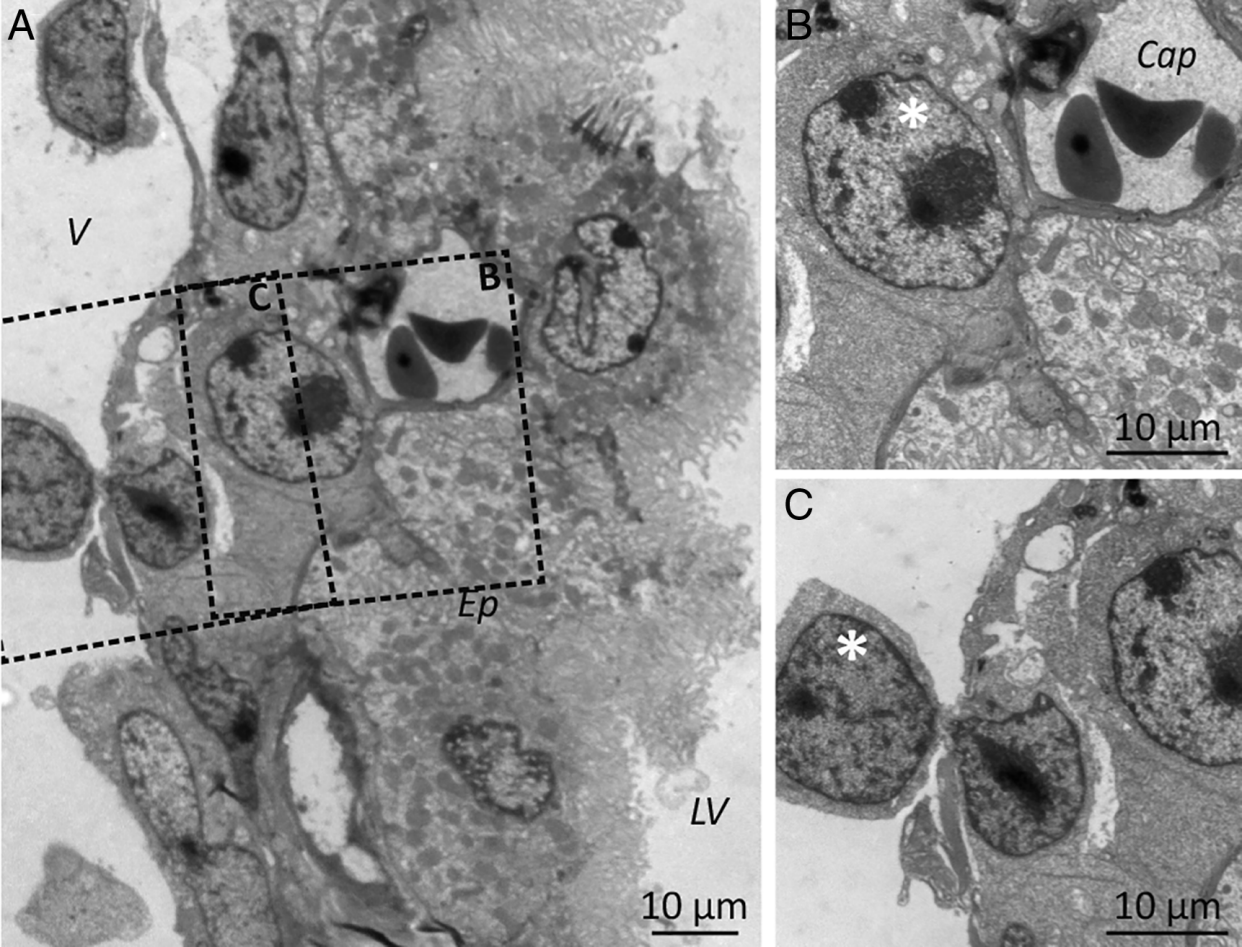
Leukaemia cells can reach the choroid plexus stroma through the choroid plexus blood vessels. (A) Electron microscopy images showing lymphoid blasts (white asterisks) in the CP stroma, both (B) in the vicinity of capillaries (Cap) and (C) crossing venule walls. V: venule; Ep: CP epithelium; LV: lateral ventricle. Samples from patients 4 and 5 were used for electron microscopy studies.

In addition, the ultrastructural studies showed that when leukaemic cells infiltrated the CNS, it was possible to observe cells with lymphoid morphology in transit between the vascular bed and the stroma in the CP (Figure 2).

### Response of choroid plexus stroma cells to the presence of leukaemic cells

To determine whether the CP stroma could provide a favourable microenvironment for BCP‐ALL cells to lodge when the CNS is invaded, we analysed the phenotypic and functional characteristics of the fibroblasts found in the CP stroma and their modification in the presence of BCP‐ALL cells.

**Figure 3:**
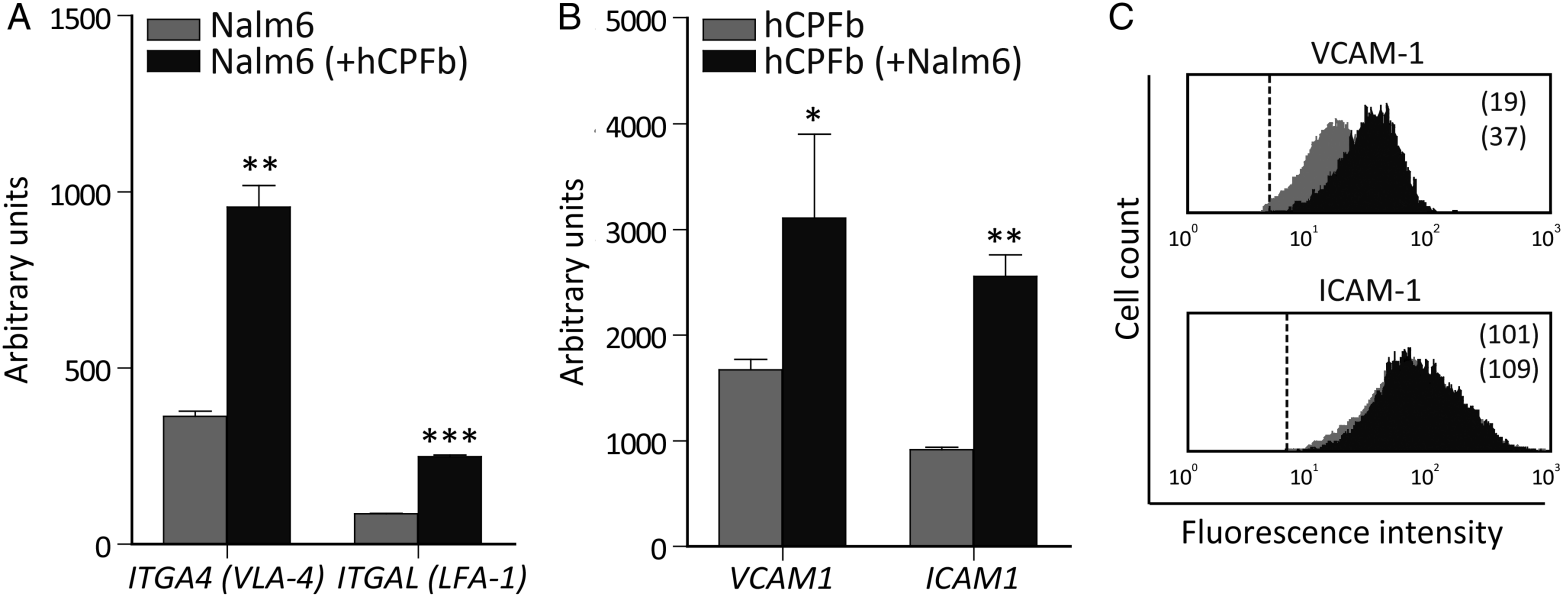
Leukaemia–choroid plexus fibroblast interaction promotes the reciprocal expression of adhesion molecules. (A) RT‐qPCR quantification of mRNA levels of *ITGA4* (VLA‐4) and *ITGAL* (LFA‐1) integrins in Nalm‐6 leukaemic cells co‐cultured for 12 h in the presence (black bars) or absence (grey bars) of human CP fibroblasts (hCPFb). (B) mRNA levels of the integrin ligands *VCAM1* and *ICAM1* in hCPFb co‐cultured with (black bars) or without (grey bars) BCP‐ALL cells. Mean ± SD of three independent experiments (*p ≤ 0.05, **p ≤ 0.01, ***p ≤ 0.001; Mann–Whitney test). (C) Representative flow cytometry histograms showing VCAM‐1 and ICAM‐1 expression in hCPFb co‐cultured in the presence (black histograms) or absence (grey histograms) of leukaemic cells. Mean fluorescence intensity values are shown.

After a co‐culture period of 24 h, most leukaemic cells appeared attached to human CP fibroblasts (supplementary material, Figure S1A). The analysis of the expression of adhesion molecules which could promote leukaemia–fibroblast interactions showed that the co‐culture of both cell types induced a significant increment in the expression levels of *ITGA4* (VLA‐4) and *ITGAL* (LFA‐1) integrins in leukaemic cells and their ligands *VCAM1* and *ICAM1* in CP fibroblasts (Figure 3A–C).

Since fibroblasts are the main cells responsible for the synthesis and remodelling of extracellular matrix in connective tissues, the expression of different extracellular matrix components was studied in CP fibroblasts cultured in the absence or presence of BCP‐ALL cells. High levels of mRNA for *COL1A1* (collagen type I), *LAMC1* (laminin), *FN1* (fibronectin), *TNC* (tenascin) as well as *HSPG2* (perlecan proteoglycan) were detected in human CP fibroblasts, and these expression levels were not altered by the presence of leukaemic cells (supplementary material, Figure S2). Similarly, no differences were observed in the expression of *MMP2*, the most abundantly expressed metalloproteinase in CP fibroblasts (supplementary material, Figure S2).

**Figure 4:**
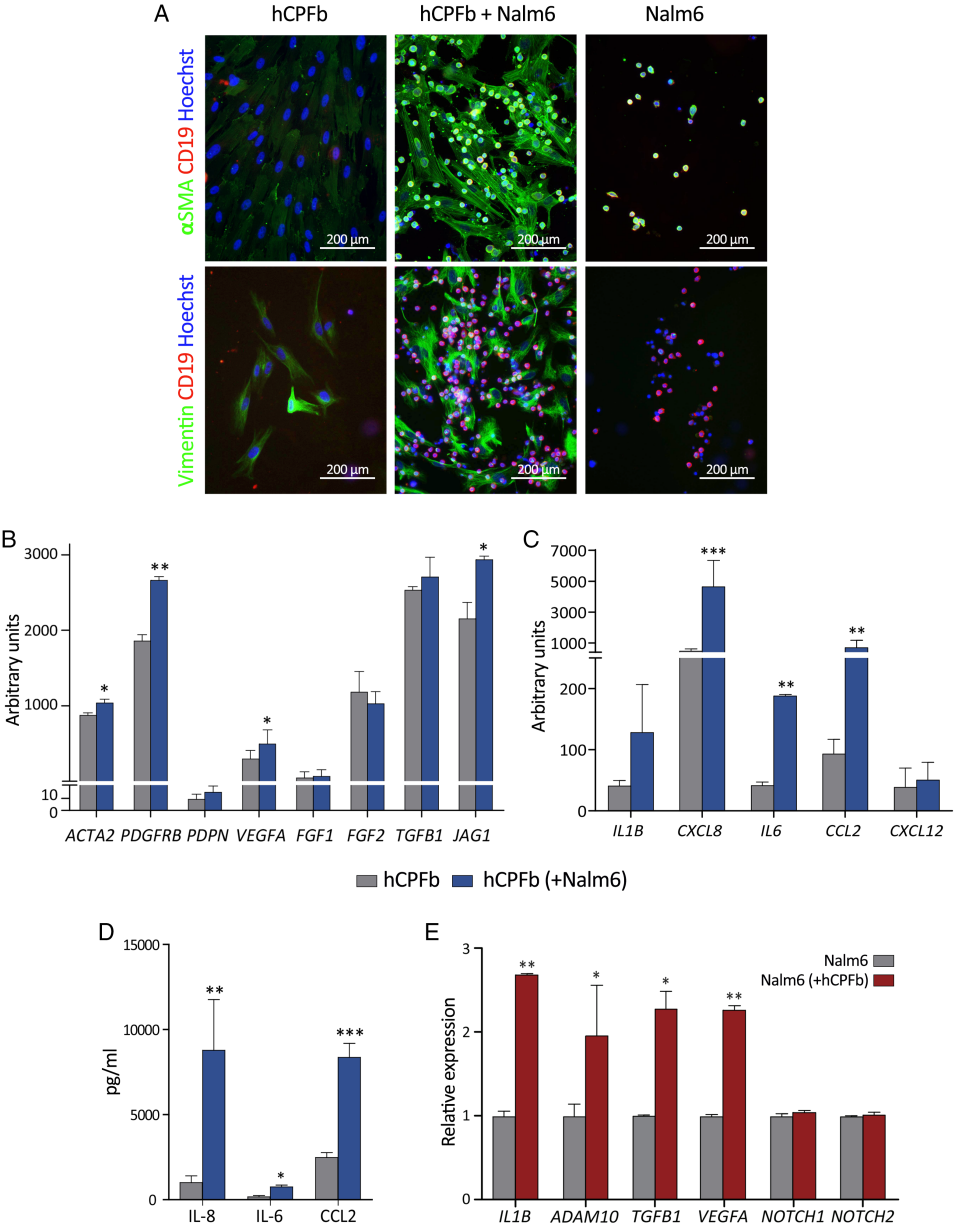
B‐cell precursor acute lymphoblastic leukaemia cells induce a cancer‐associated fibroblast (CAF) phenotype in human choroid plexus fibroblasts. (A) Analysis of α‐SMA and vimentin expression (green staining) in human choroid plexus fibroblasts (hCPFb) co‐cultured for 12 h in direct contact with or without Nalm‐6 cells (red staining). (B) RT‐qPCR quantification of mRNA levels for different CAF markers, as well as the Notch ligand *JAG1* (Jagged1), in hCPFb cultured for 12 h in the presence (blue bars) or absence (grey bars) of BCP‐ALL cells in a Transwell system. (C) mRNA expression levels for different cytokines and chemokines in hCPFb co‐cultured with (blue bars) or without (grey bars) BCP‐ALL cells. (D) The concentrations of IL‐8, IL‐6, and CCL2 in supernatants collected from leukaemia–CP fibroblast co‐cultures after 72 h were determined by ELISA and CBA systems. (E) Changes in the mRNA expression profile of Nalm‐6 leukaemic cells co‐cultured in the presence (red bars) or absence (grey bars) of hCPFb, assessed using RT‐qPCR. Mean ± SD of three independent experiments (*p ≤ 0.05, **p ≤ 0.01, ***p ≤ 0.001; Mann–Whitney test).

Using the same co‐culture system, we analysed the acquisition of cancer‐associated fibroblast (CAF) markers by CP fibroblasts in response to the presence of leukaemic cells. Higher expression of α‐SMA, accompanied by enhanced expression of vimentin, was found in those CP fibroblasts cultured with leukaemic cells (Figure 4A,B). In addition, increased expression levels of the CAF markers *PDGFRB*, *PDPN* (podoplanin), and *VEGFA* were observed when CP fibroblasts had been cultured with leukaemic blasts. No changes were detected in the expression of *FGF1/2* or *TGFB1* (Figure 4B). After contact with BCP‐ALL cells, CP fibroblasts also upregulated the mRNA and protein expression levels of pro‐inflammatory cytokines and chemokines, such as IL‐6, CCL2, and mainly IL‐8 (Figure 4C,D).

On the other hand, the analysis of leukaemia cell/CP fibroblast co‐cultures also showed that CD19^+^ blasts expressed *IL1B*, *TGFB1*, *VEGFA*, and the metalloproteinase *ADAM10*, and this expression was significantly upregulated in the presence of CP fibroblasts (Figure 4E). No expression of *IL6*, *IL8* or *CCL2* mRNA was detectable by RT‐PCR. Since Notch signalling has been involved in B‐cell ALL [19], the expression of Notch receptors was analysed, showing that leukaemic cells expressed detectable levels of *NOTCH1* and *NOTCH2* (but not *NOTCH3* or *NOTCH4*) receptors, which were not influenced by the co‐culture with CP fibroblasts (Figure 4E).

### The choroid plexus stroma acts as a sanctuary for leukaemic cells

**Figure 5:**
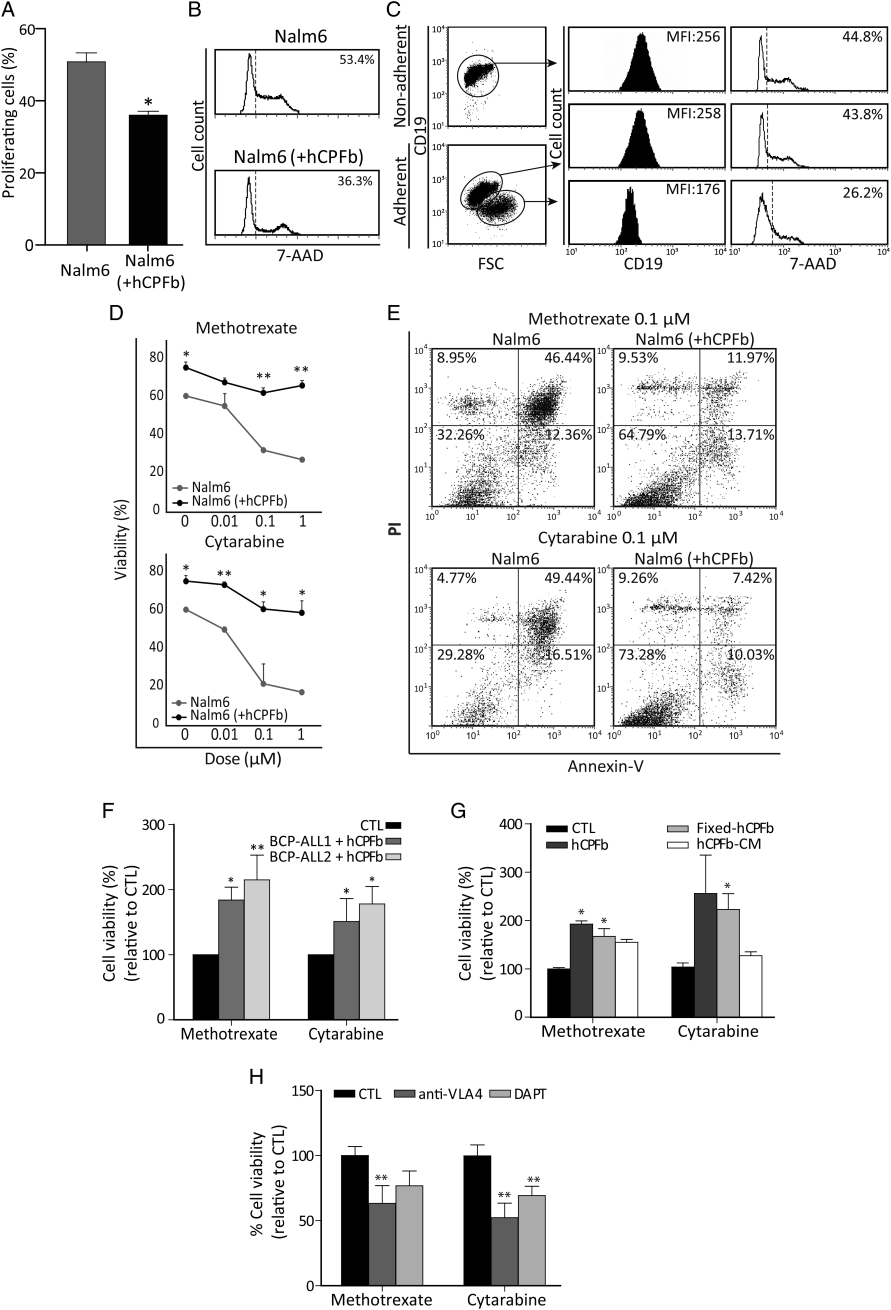
Human choroid plexus fibroblasts protect leukaemia cells from chemotherapy through VCAM‐1/VLA‐4 interactions and Notch signalling. (A) The proliferation rate of Nalm‐6 cells cultured in the presence or absence of human CP fibroblasts (hCPFb) for 48 h was determined by 7‐AAD staining and analysed by flow cytometry. Mean ± SD of proliferating cells from three independent experiments. (B) Representative flow cytometry histograms showing the fraction of cells in S + G2 + M phases. (C) Analysis of leukaemic cell size and CD19 expression when co‐cultured with hCPFb. Dot plots show forward scatter (FSC) properties and CD19 expression of Nalm‐6 cells from non‐adherent (in suspension) and adherent (attached to fibroblasts) fractions. Representative flow cytometry histograms show CD19 expression (solid black histograms) and 7‐AAD staining (open histograms). Mean fluorescence intensity (MFI) and percentage of proliferating cells, respectively, are indicated. (D) The cell viability of Nalm‐6 leukaemic cells exposed to increasing concentrations (0.01, 0.1, and 1 μm) of chemotherapeutic agents (methotrexate and cytarabine) in the presence (black lines) or absence (grey lines) of hCPFb for 72 h was assessed by flow cytometry. Results show the percentage of propidium iodide‐negative and annexin V‐negative viable leukaemic cells, and are the mean ± SD of four independent experiments. (E) Representative dot plots showing the viability of leukaemic cells treated with methotrexate and cytarabine (0.1 μm) in the presence of hCPFb or in control cultures analysed by annexin V and propidium iodide staining. (F) Primary samples from two BCP‐ALL patients (Nos 1 and 2) were exposed to methotrexate and cytarabine (0.1 μm) in the presence or absence of hCPFb. Results from three independent experiments are expressed as the mean ± SD of the relative viability compared with control cultures performed in the absence of hCPFb. (G) Relative viability of Nalm‐6 leukaemic cells co‐cultured with hCPFb, paraformaldehyde‐fixed hCPFb or cultured in the presence of hCPFb‐derived conditioned media and treated with methotrexate and cytarabine at a concentration of 0.1 μm. Data from three independent experiments are expressed as the mean ± SD of the relative viability compared with Nalm‐6 cell control cultures. (H) Cell viability of Nalm‐6 cells treated with chemotherapeutic agents and co‐cultured with hCPFb in the presence of anti‐VLA‐4 blocking antibodies or the Notch inhibitor, DAPT, compared with control cultures (CTL: anti‐IgG or DMSO). Results represent the mean ± SD of three to four independent experiments (*p ≤ 0.05, **p ≤ 0.01; Mann–Whitney test).

In order to know whether CP fibroblasts had the ability to induce BCP‐ALL cell chemoresistance, the proliferative rate of leukaemic cells grown in co‐culture was analysed. As shown in Figure 5A,B, the presence of CP fibroblasts induced a significant decrease in the proliferation of leukaemia cells. This effect was particularly evident in a subset of large‐sized leukaemic cells (15–20% of the whole cell population) which exhibited low expression levels of CD19 and appeared firmly attached to CP fibroblasts (Figure 5C and supplementary material, Figure S1B).

The effects of CP fibroblasts on the viability of leukaemic cells cultured with increasing doses of chemotherapy drugs commonly used in the treatment of childhood BCP‐ALL were next analysed. BCP‐ALL cell viability was always significantly higher in those cultures established with CP fibroblasts (Figure 5D–F) and, remarkably, this survival‐promoting effect could be observed even upon 72 h of culture with the highest level (1 μm) of either methotrexate or cytarabine (Figure 5D). On the other hand, the viability of CP fibroblasts was not affected by the chemotherapeutic agents (supplementary material, Figure S3).

To investigate the mechanisms contributing to the chemoresistance of BCP‐ALL cells in contact with CP fibroblasts, leukaemic blasts were treated with methotrexate or cytarabine and their viability was determined in the absence or presence of live and fixed CP fibroblasts or their conditioned medium (Figure 5G). Although both physical interactions and soluble factors participated in CP fibroblast‐mediated chemoprotection, the involvement of cell‐to‐cell contacts seemed to be more relevant (Figure 5G).

Given the increased expression of *ITGA4* (VLA‐4) and its ligand *VCAM‐1* after leukaemia/CP fibroblast co‐culture (Figure 3) and the relevance of the interactions between VLA‐4 on leukaemic cells and VCAM‐1 on bone marrow stromal cells in promoting adhesion and chemoresistance [20], we used anti‐VLA‐4 antibodies to study the effects of the blockade of VLA‐4/VCAM‐1 interaction on CP fibroblast‐induced chemoprotection. Figure 5H shows that the addition of anti‐VLA‐4 antibodies to the co‐cultures significantly reduced the chemoprotective properties of CP fibroblasts, decreasing by 40–50% the viability of BCP‐ALL cells.

Likewise, we also analysed the effects of blocking the Notch signalling pathway since Notch inhibitors seem to enhance B‐cell ALL chemosensitivity [19], and our results showed that in co‐culture CP fibroblasts upregulated the expression of the Notch ligand *JAG1* (Jagged‐1) (Figure 4B) and leukaemic cells expressed Notch receptors and increased levels of *ADAM10*, an essential protease for Notch pathway activation (Figure 4E). Thus, leukaemia/CP fibroblast co‐cultures were set up in the presence or absence of the DAPT inhibitor and the results showed that after 72 h, leukaemia cell viability was reduced by 25–35% when Notch signalling was inhibited (Figure 5H).

## Discussion

CNS leukaemia is considered to be primarily a leptomeningeal disease [12, 21, 22] and the BCSFB, formed by the epithelial lining of the CP, has been pointed out by several authors to play a key role in ALL cell dissemination to the leptomeninges [23, 24, 25]. However, the pathway by which leukaemic blasts could reach the BCSFB has not been defined yet. In this regard, it is interesting to note that the CP is involved in the immune surveillance of the CNS and that the CP vasculature, with its fenestrated endothelium, has been described to constitute a gateway for immune cell entry into the brain, both in health and in disease [17, 26, 27]. Our results with a xenograft model using primary BCP‐ALL cells show that CD19^+^ leukaemic cells could be detected attached to the luminal surface of the CP epithelium in the brain ventricles but also forming cell clusters in the connective tissue stroma of the CP. The electron microscopy results corroborated the presence of lymphoid blasts in this CP compartment and also show, in an *in vivo* model, the traffic of leukaemia cells between the bloodstream and the connective tissue axis of the CP. BCP‐ALL cell migration from blood could be facilitated by the intrinsic higher permeability properties of the CP fenestrated capillaries but also by the possible modifications that endothelial cells could undergo in response to the presence of leukaemia cells [22, 28].

Once the CP stroma is reached, leukaemia cells can have two fates: to stay in the connective stroma or to migrate across the CP epithelium towards the CSF. As commented above, ALL cell lines have been described as able to cross CP epithelial cell monolayers [24, 25]; however, in our immunofluorescence and electron microscopy studies, only exceptionally did we find leukaemic cells in transit from the basal to the apical surface of the CP epithelium, suggesting that the BCSFB is a minor pathway for leptomeningeal dissemination, as has also been pointed out by other authors [5, 29]. Supporting this, we were not able to detect expression of the complement component C3 in BCP‐ALL cells (data not shown), in contrast to leptomeningeal metastatic cells from breast and lung cancer that secrete C3 to activate C3a receptor in CP epithelial cells and disrupt the BCSFB, allowing molecules and cells to enter the CSF [30]. With this in mind, it is likely that most leukaemia cells reaching the CP stroma stay there since they could find a permissive microenvironment for their maintenance. Using bone marrow chimeric mice in which the recipient's immune system was reconstituted with GFP‐transgenic bone marrow cells, Hasegawa‐Ishii *et al* [31] showed in the CP stroma the presence of marrow‐derived CXCL12‐expressing fibroblastic cells, similar to those CAR (CXCL12‐abundant reticular) cells which play an important role in B‐cell development in the bone marrow [32]. These data point out that the CP stroma could serve as a niche for haematopoietic precursor cells, which is in agreement with other findings showing the existence of nestin‐positive fibroblastic cells closely associated to myeloid progenitors in the choroidal stroma [33], and its potential to support extramedullary haematopoiesis (our unpublished observations) [34]. Also in support, our data show that ALL cells strongly adhere to CP fibroblasts, which is promoted by the upregulation of the expression of *ITGA4* (VLA‐4) and *ITGAL* (LFA‐1) integrins in leukaemic cells and their corresponding ligands, *VCAM1* and *ICAM1*, in CP fibroblasts. In addition, some BCP‐ALL cells are also able to migrate beneath the layer of CP fibroblasts, a process termed pseudo‐emperipolesis usually seen in long‐term Whitlock–Witte cultures supporting B‐lymphopoiesis [35].

Apart from providing a proper microenvironment for the maintenance of haematopoietic precursor cells, the CP stroma could be further modified by leukaemia cells. Previous work has reported that BCP‐ALL cells, like other types of tumour cells, have the capacity to induce a CAF phenotype in bone marrow fibroblastic‐like cells [36, 37]. CAFs have been described as key components of the tumour microenvironment and appear in close contact with cancer cells, exhibiting particular phenotypic and functional characteristics, induced upon interaction with tumour cells, and promoting tumour growth and survival [38, 39]. Our present results show that BCP‐ALL cells are also able to modify the CP stroma's cell components and induce a CAF phenotype characterized by the upregulated expression of α‐SMA and vimentin, as well as pro‐inflammatory factors IL‐6, IL‐8, and CCL2. These data agree with earlier studies which showed a pro‐inflammatory profile in bone marrow mesenchymal stem cells derived from BCP‐ALL patients [40, 41].

An important result from our study is that CP fibroblasts have the capacity to induce chemoresistance to methotrexate and cytarabine, both drugs commonly used in ALL therapy. Several non‐exclusive mechanisms could be mediating this chemoprotective effect of the CP stroma to promote leukaemia cell survival. Soluble factors such as IL‐6 and IL‐8, whose expression notably increases upon leukaemia–CP fibroblast interaction, have been recently described as responsible for CAF‐induced chemoresistance in breast and lung cancer cells [42]. However, de Vasconcellos *et al* [43] pointed out that those cytokines would not play a direct role in B‐cell ALL cells but would modify microenvironment cell components to favour leukaemia progression. Therefore, cell–cell contacts seem to be a more determinant factor in the protection of leukaemic cells by stromal cell components. In this regard, VLA‐4/VCAM‐1 interactions have been involved in the induction of chemoresistance in B‐cell ALL [20, 44] as well as other haematological malignancies [45, 46], and our data show that, as in bone marrow, the blockade of VLA‐4 signalling in BCP‐ALL cells is also able to partially revert the chemoresistance induced by CP fibroblasts. Another signalling pathway involved in the acquisition of resistance to chemotherapy is the Notch pathway, whose involvement in T‐cell ALL has been described repeatedly [47, 48], but its role in B‐cell ALL is starting to be elucidated. Takam Kamga *et al* [19] have recently shown, using a human B‐cell ALL xenograft mouse model, that the administration of a Notch inhibitor potentiated the reduction of the bone marrow leukaemic burden induced by the chemotherapeutic agent cytarabine, indicating that Notch signalling participates in the acquisition of chemoresistance by ALL cells. Similarly, our results showing the upregulated expression of *JAG1* (Jagged‐1) and *ADAM‐10* in CP fibroblasts and BCP‐ALL cells, respectively, and the increased chemosensitivity of leukaemic cells after treatment with a γ‐secretase inhibitor, indicate that the activation of the Notch pathway occurring after CP fibroblast–leukaemia cell interaction is also partially responsible for the resistance to chemotherapy acquired by BCP‐ALL cells.

**Figure 6:**
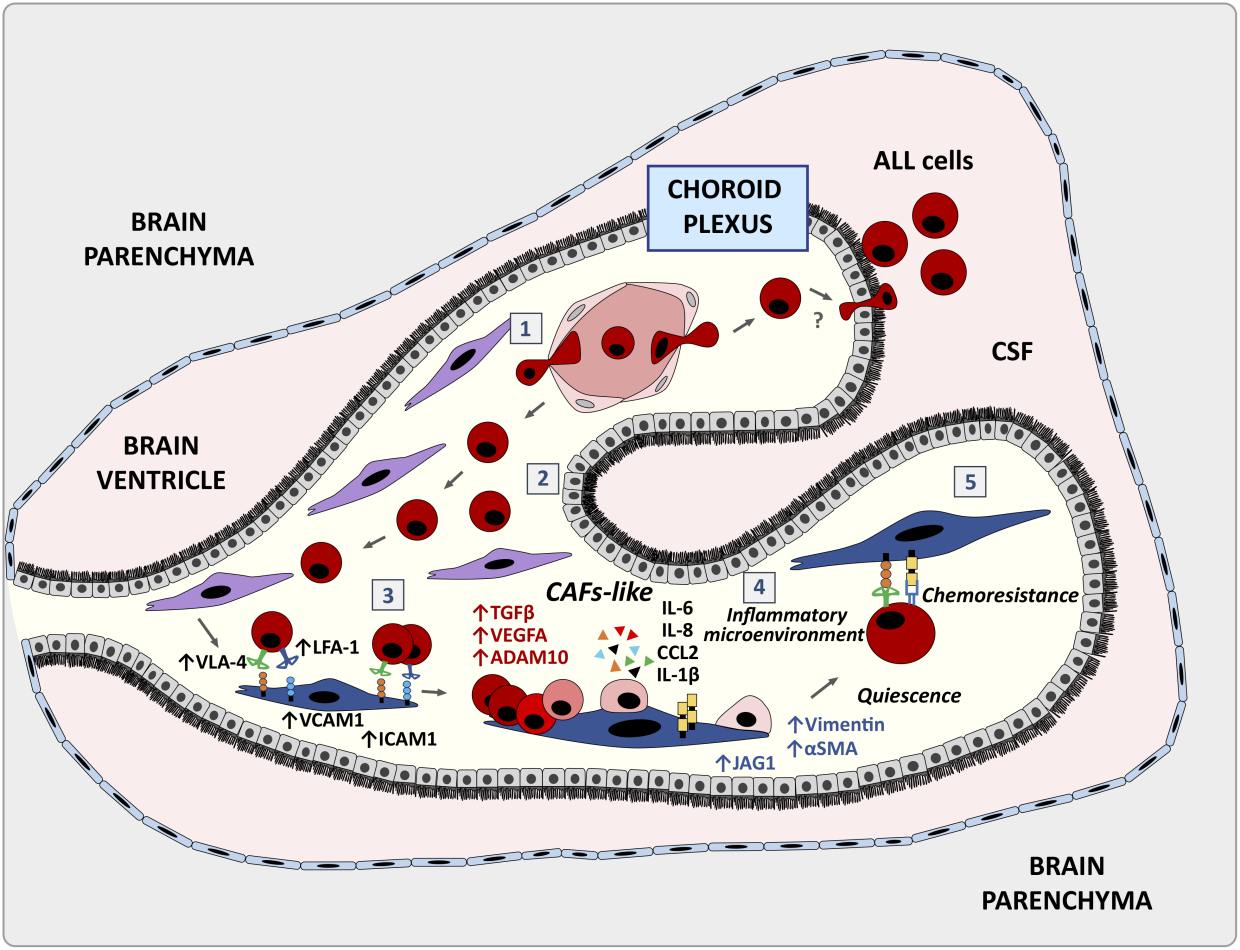
The choroid plexus stroma could act as a sanctuary for B‐cell precursor acute lymphoblastic leukaemia cells. Schematic representation of the involvement of the choroid plexus in acute lymphoblastic leukaemia: BCP‐ALL cells could reach the CP via its fenestrated vasculature that can become more permissive under the influence of leukaemic cells (1). Once the CP is colonized, BCP‐ALL cells would preferentially stay in the connective stroma and occasionally move towards the CSF crossing the CP epithelial lining which forms the BCSFB (2). BCP‐ALL cells would then interact with the CP stromal fibroblasts through the upregulated expression of VCAM‐1/VLA‐4 and ICAM‐1/LFA‐1 ligand–receptor pairs (3) and induce a CAF phenotype which would promote a pro‐tumoural inflammatory microenvironment (4). BCP‐ALL cells would remain attached to the CP CAF‐like cells and could acquire quiescence and chemoresistance, a process partially dependent on the VLA‐4 and Notch signalling pathways (5).

Taken together, we propose that the CP stroma may represent a sanctuary for paediatric BCP‐ALL cells (Figure 6). Leukaemic cells would not be actively recruited by specific chemoattractant signals but would arrive at this CNS location due to its increased vascular permissiveness. Other brain areas containing fenestrated capillaries, such as the circumventricular organs (including the area postrema, subfornical organ, organum vasculosum of the lamina terminalis, median eminence, neurohypophysis, and pineal gland) can also be infiltrated by ALL cells [5], supporting the idea of a permissive entry to these sites. Furthermore, extravasation into the CP stroma could be favoured by modifications in vascular adhesion molecule expression induced by BCP‐ALL cells. Once in the CP stroma, leukaemia blasts would occasionally cross the CP epithelium forming the BCFSB or, more likely, stay in the CP stroma where they find a haematopoiesis‐supporting microenvironment that they could further modify. Changes in CP stromal cell components could induce a reduction in the proliferation of leukaemia cells which would facilitate the acquisition of chemoresistance to drugs conventionally used in ALL therapy. These chemoresistant BCP‐ALL cells could then persist in the CP as minimal residual disease and be in part responsible for later relapses. In this regard, it could be hypothesized that at some moment quiescent BCP‐ALL cells could escape from dormancy and reach either the ventricular cavities by crossing the epithelial BCSFB or the subarachnoid space directly by migrating through the loose connective tissue of the tela choroidea, a very thin region of meningeal pia mater, and underlying ependymal, which is in continuity with the CP stroma in the medial wall of the lateral ventricles and the roof of the third and fourth ventricles.

## Author contributions statement

LMFS, JV, MAFV, AGM, RS and EJ performed research. AGM and MR provided patient samples and clinical information. AVi, AVa and MR supervised the study. LMFS, AVa and AVi designed experiments, analysed, and interpreted data, and wrote the manuscript. All the authors agreed with the submission in its final form.

## Supporting information


**Figure S1.** BCP‐ALL cells attach strongly to choroid plexus fibroblasts
**Figure S2.** Expression of extracellular matrix components in choroid plexus fibroblasts
**Figure S3.** Chemotherapeutic agents do not affect the viability of choroid plexus fibroblasts

**Table S1.** Characteristics of BCP‐ALL patients at diagnosis included in this study
**Table S2.** TaqMan gene expression assays used
**Table S3.** Antibodies used in this study
