# Supplementary material for: The choroid plexus stroma constitutes a sanctuary for paediatric B‐cell precursor acute lymphoblastic leukaemia in the central nervous system
Source: J Pathol. 2020 Aug 28;252(2):189–200. doi: 10.1002/path.5510 (PMC7540040; doi:10.1002/path.5510)
Supplement: Supplementary file 1 — Figure S1. BCP‐ALL cells attach strongly to choroid plexus fibroblasts Figure S2. Expression of extracellular matrix components in choroid plexus fibroblasts Figure S3. Chemotherapeutic agents do not affect the viability of choroid plexus fibroblasts [file PATH-252-189-s001.docx]

**The choroid plexus stroma constitutes a sanctuary for paediatric B-cell precursor acute lymphoblastic leukaemia in the central nervous system**

LM Fernández-Sevilla *et al. J Pathol* DOI: 10.1002/path.5510

**Supplementary Figures S1–S3**


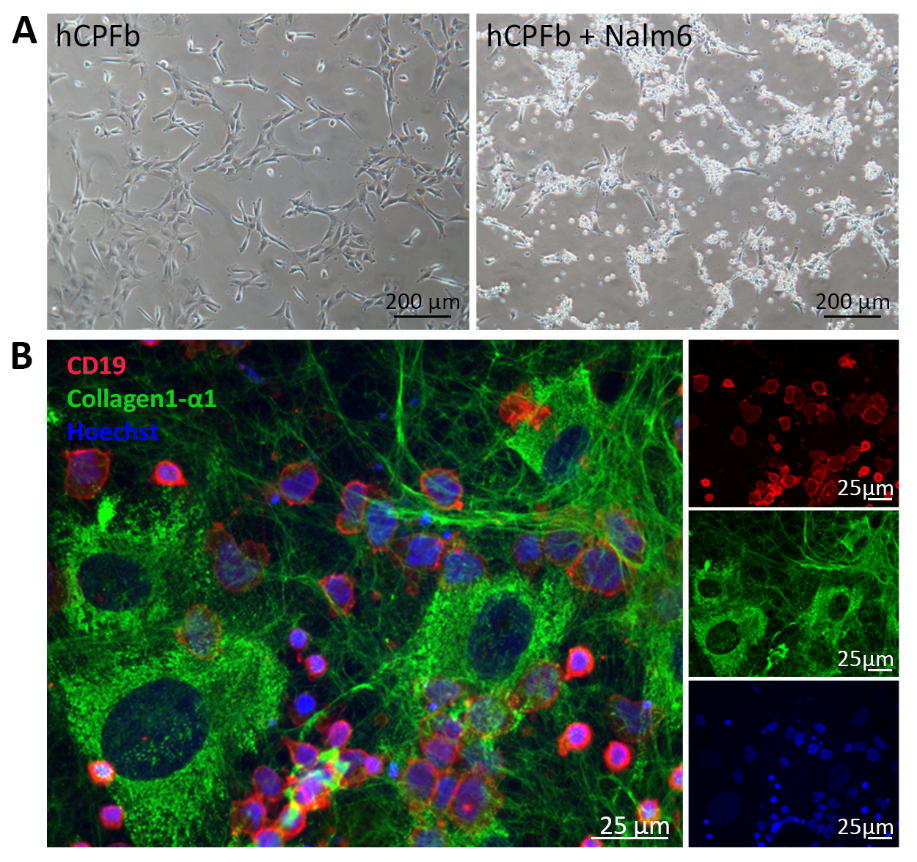


**Figure S1.** BCP-ALL cells attach strongly to choroid plexus fibroblasts. (A) Representative images of human CP fibroblasts (hCPFb) cultured alone or with Nalm-6 leukaemic cells for 24 h. (B) Fluorescence microscopy images of leukaemic cells (CD19 staining, red) co-cultured with hCPFb (type I collagen staining, green) for 72 h. Cell nuclei were counterstained with Hoechst 33258.





**Figure S2.** Expression of extracellular matrix components in choroid plexus fibroblasts. RT-qPCR measurement of mRNA levels in human CP fibroblasts (hCPFb) seeded in the lower side of a Transwell insert and cultured for 12 h in the absence (grey bars) or presence (white bars) of Nalm-6 leukaemic cells on the upper side of the insert. Mean ± SD of three or four independent experiments. *COL1A1*: encoding the α1 chain of type I collagen; *LAMC1*: encoding the laminin γ1 chain; *FN1*: encoding fibronectin; *TNC*: encoding tenascin; *HSPG2*: encoding heparan sulphate proteoglycan 2/perlecan; *MMP2*: encoding matrix metalloproteinase 2.





**Figure S3.** Chemotherapeutic agents do not affect the viability of choroid plexus fibroblasts. Primary human CP fibroblasts (hCPFb) were treated for 72 h with increasing concentrations of methotrexate and cytarabine (0.01, 0.1, and 1 µm), and then viability was assessed by flow cytometry. Mean ± SD of the percentage of viable cells from three independent experiments.
