# Supplementary material for: The choroid plexus stroma constitutes a sanctuary for paediatric B‐cell precursor acute lymphoblastic leukaemia in the central nervous system
Source: J Pathol. 2020 Aug 28;252(2):189–200. doi: 10.1002/path.5510 (PMC7540040; doi:10.1002/path.5510)
Supplement: Supplementary file 2 — Table S1. Characteristics of BCP‐ALL patients at diagnosis included in this study Table S2. TaqMan gene expression assays used Table S3. Antibodies used in this study [file PATH-252-189-s002.docx]

**The choroid plexus stroma constitutes a sanctuary for paediatric B-cell precursor acute lymphoblastic leukaemia in the central nervous system**

LM Fernández-Sevilla *et al. J Pathol* DOI: 10.1002/path.5510

**Supplementary Tables S1–S3**

**Table S1.** Characteristics of BCP-ALL patients at diagnosis included in this study. BCP-ALL patients’ stratification into high or non-high (low/intermediate) risk of relapse and CNS disease (presence of leukaemic blasts in CSF) were defined according to SEHOP-PETHEMA 2013 (Spanish Program for the Treatment of Hematologic Diseases). Minimal residual disease (MRD) evaluation: +33 days

| **ID** | **Phenotype** | **Sex** | **Age at diagnosis (years)** | **% Blasts** | **Risk of relapse** | **CNS disease** | **MRD** | **Relapse** | **Site of relapse** | **DFS (days)** |
| --- | --- | --- | --- | --- | --- | --- | --- | --- | --- | --- |
| 1 | B | F | 0.8 | 90 | High risk | − | + | + | BM | 280 |
| 2 | B | F | 6 | 90 | Non-high risk | − | − | − |  | 2592 |
| 3 | B | M | 0.2 | 90 | High risk | − | − | − |  | 2920 |
| 4 | B | M | 10 | 95 | High risk | − | + | − |  | 2555 |
| 5 | B | M | 3 | 90 | Non-high risk | − | − | − |  | 2957 |
| 6 | B | M | 8.6 | 90 | Non-high risk | − | − | − |  | 1280 |
| 7 | B | M | 9.7 | 95 | Non-high risk | − | − | − |  | 1277 |
| 8 | B | F | 3.5 | 90 | Non-high risk | − | − | − |  | 1095 |
| 9 | B | M | 3.7 | 95 | Non-high risk | − | − | − |  | 1058 |
| 10 | B | M | 7.1 | 95 | Non-high risk | − | − | − |  | 620 |
| 11 | B | M | 7 | 90 | − | − | − | + | CNS/BM | 1004 |

BM, bone marrow; DFS, disease-free survival.

**Table S2.** TaqMan gene expression assays used

| **Gene symbol** | **Reference** | **Gene symbol** | **Reference** |
| --- | --- | --- | --- |
| *ACTA2* | Hs00426835_g1 | *ITGA4* | Hs00168433_m1 |
| *ADAM10* | [Hs00153853_m1](https://www.thermofisher.com/taqman-gene-expression/product/Hs00153853_m1?CID=&ICID=&subtype=) | *ITGAL* | Hs00164957_m1 |
| *CCL2* | Hs00234140_m1 | *JAG1* | Hs01070032_m1 |
| *COL1A1* | Hs00164004_m1 | *LAMC1* | Hs00267056_m1 |
| *CXCL12* | Hs00171022_1m1 | *MMP2* | Hs01548727_m1 |
| *CXCL8* | Hs00174103_m1 | *NOTCH1* | Hs01062014_m1 |
| *FGF1* | Hs01092738_m1 | *NOTCH2* | Hs01050702_m1 |
| *FGF2* | Hs00266645_m1 | *PDGFRB* | Hs01019589_m1 |
| *FN1* | Hs01549976_m1 | *PDPN* | Hs00366766_m1 |
| *GNB2L1* | Hs00272002_m1 | *TGFB1* | Hs00998133_m1 |
| *HSPG2* | Hs01078536_m1 | *TNC* | [Hs01115665_m1](https://www.thermofisher.com/taqman-gene-expression/product/Hs01115665_m1?CID=&ICID=&subtype=) |
| *ICAM1* | HS00164932_m1 | *VCAM1* | Hs01003372_m1 |
| *IL1B* | Hs01555410_m1 | *VEGFA* | Hs00900055_m1 |
| *IL6* | Hs00985639_m1 |  |  |

**Table S3.** Antibodies used in this study

| **Antigen** | **Clone** | **Dilution** | **Supplier** | **Reference** |
| --- | --- | --- | --- | --- |
| CD19 | A3-B1 | 1:100 | ImmunoStep, Salamanca, Spain | 19PU-01MG |
| CD19 | HIB19 | 1:60 | BioLegend, San Diego, CA, USA | 302206 |
| CD45.1 | A-20 | 1:100 | BioLegend | 110708 |
| Pan-cytokeratin | C11 | 1:400 | Sigma-Aldrich, St Louis, MO, USA | F3418 |
| CD106 (VCAM1) | 51-10C9 | 1:20 | BD Biosciences, San José, CA, USA | 551147 |
| CD54 (ICAM1) | HCD54 | 1:20 | BioLegend | 322714 |
| α-Smooth muscle actin (α-SMA) | EPR5368 | 1:100 | Merck, Darmstadt, Germany | MABT381 |
| Vimentin | D21H3 | 1:100 | Cell Signaling Technology, Leiden, The Netherlands | 5741 |
| Collagen type 1 α1 | Polyclonal | 1:100 | Sigma-Aldrich | HPA011795 |
